# IFN-γ, IL-2, IP-10, and MIG as Biomarkers of Exposure to *Leishmania* spp., and of Cure in Human Visceral Leishmaniasis

**DOI:** 10.3389/fcimb.2017.00200

**Published:** 2017-05-31

**Authors:** Ana V. Ibarra-Meneses, Prakash Ghosh, Faria Hossain, Rajashree Chowdhury, Dinesh Mondal, Jorge Alvar, Javier Moreno, Eugenia Carrillo

**Affiliations:** ^1^WHO Collaborating Centre for Leishmaniasis, Centro Nacional de Microbiologia, Instituto de Salud CarlosMadrid, Spain; ^2^Nutrition and Clinical Services Division, International Centre for Diarrhoeal Disease ResearchDhaka, Bangladesh; ^3^Drugs for Neglected Diseases InitiativeGeneva, Switzerland

**Keywords:** chemokine, cytokine, biomarker, leishmaniasis, whole blood assay, asymptomatic, treatment

## Abstract

New biomarkers are needed for monitoring the effectiveness of treatment for visceral leishmaniasis (VL). They might also improve the detection of the asymptomatic population in *Leishmania-*endemic areas. This paper examines the IL-2, IFN-γ, IFN-γ-induced protein 10 (IP-10), and monokine-induced-by-IFN-γ (MIG) levels in whole blood—stimulated *in vitro* with soluble *Leishmania* antigen (SLA)—taken from asymptomatic individuals and patients treated for VL living in a post-outbreak (*Leishmania infantum*) area in Spain, and in an endemic (*Leishmania donovani*) area of Bangladesh. IP-10 was found to be an accurate global marker of asymptomatic subjects with positive cellular/humoral tests, while MIG was found to be a better marker of contact with *L. donovani* than IL-2 but no for those with *L. infantum*. Determining IP-10, MIG, and IFN-γ levels proved useful in monitoring the cellular immune response following treatment for active disease caused by *L. infantum*.

## Introduction

Visceral leishmaniasis (VL), or kala-azar, is one of the deadliest and most neglected of all tropical diseases. Effective therapy is central to any strategy for controlling leishmaniasis, and biomarkers able to indicate the initiation of a successful response might help shorten the duration of treatment. The identification and management of asymptomatic subjects has become an increasingly important challenge in VL control programs, yet no validated markers of asymptomatic infection exist, and the parasitological, molecular and serological tools currently used are not entirely suitable (Vallur et al., 2016). There is a therefore a pressing need for new biomarkers that can identify the asymptomatic population in areas where *Leishmania* is endemic, and that can aid in monitoring the success of treatment in patients with active disease.

IFN-γ in soluble *Leishmania* antigen (SLA)-stimulated whole blood (*in vitro*) has recently been described to gradually but significantly increase after the successful treatment of patients infected with *Leishmania donovani* (Adem et al., 2016) and *Leishmania infantum* (Ibarra-Meneses et al., 2016). However, this assay has never been used to monitor individual patients from the time of their having active disease through to their cure. IFN-γ and IL-2 concentrations following whole blood stimulation with SLA have also been shown to provide accurate markers of asymptomatic *L. donovani* (Gidwani et al., 2011) and *L. infantum* infection (Ibarra-Meneses et al., 2016). However, despite their high sensitivity and sensitivity, IFN-γ and IL-2 are only produced in small amounts; more robust biomarkers would be preferable.

Interest has recently grown in the use of chemokines as alternative biomarkers. When activated by IFN-γ, many cell types produce IFN-γ-inducible protein-10 (IP-10) and monokine-induced-by-IFN-γ (MIG; Gasperini et al., 1999), which further increase the production of IFN-γ. Both chemokines provide accurate biomarkers of a range of infections, including those of *Mycobacterium tuberculosis*, hepatitis C virus and malaria parasites (Azzurri et al., 2005; Armah et al., 2007; Falconer et al., 2010). In recent years, chemokines have been identified in the host response against *Leishmania* (Oghumu et al., 2010). Indeed, IP-10 has been shown critical in the induction of cellular immunity following vaccination against *L. donovani* (Fallahi et al., 2016), and to play a protective role in reducing the number of intracellular parasites in cutaneous lesions (Vasquez and Soong, 2006). Further, plasma MIG and IP-10 have been shown to increase in patients treated for *L. donovani*-induced VL (Hailu et al., 2004). These chemokines might therefore provide a sensitive means of detecting antigen-specific T-cell responses following *Leishmania* infection.

The present work examines IP-10 and MIG concentrations in SLA-stimulated whole blood as potential markers of asymptomatic individuals with *L. infantum*/*L. donovani* infection. The usefulness of these chemokines and of the cytokines IFN-γ and IL-2 in monitoring the cellular immune response, and therefore as markers of cure in patients treated for VL, was also investigated.

## Materials and methods

### Data collection and participants

Three hundred and five blood donors attending the *Hospital de Fuenlabrada* Blood Bank, plus 12 patients with active VL and another 14 cured patients (all cared for by the same hospital's Internal Medicine Department), were recruited over 2015-2016. All were aged ≥18 years and resided in the town of Fuenlabrada (previously described as a focus of *L. infantum* transmission). Blood samples from all the above were subjected to peripheral blood mononuclear cell isolation and to the *in vitro* cell proliferation assay (CPA), performed as previously described (Ibarra-Meneses et al., 2016). Of the 305 blood bank samples, 57 were CPA-positive (stimulation index ≥2.27); the subjects providing these samples were thus considered asymptomatic (AS-Li) and their blood was selected for further analysis of cytokines and chemokines. Fifty CPA-negative samples were randomly selected as negative endemic *L. infantum* controls (NVL-Li). Six of the 12 patients with active VL, all treated with liposomal amphotericin B (WHO, 2010), underwent medical examination at 0, 3, 6, and 12 after the start treatment, at which times their blood was also sampled. Following WHO guidelines, treated patients were considered cured (CVL) if they showed no relapse by 6 months after beginning treatment (WHO, 2010)—a criterion met by all the present treated patients.

In October 2016, 25 healthy subjects, all ≥18 years of age, were also recruited from Mymensingh in Bangladesh, an area endemic for *L. donovani-*induced VL. Of these subjects, 12 were classified as asymptomatic (AS-Ld) [based on the rK39 and direct agglutination (DAT) tests] and 13 as negative endemic *L. donovani* controls (NVL-Ld). Table 1 shows the clinical characteristics of the final study subjects.

**Table 1:** **Clinical characteristics of the study population**.

**Group**	**No. of subjects**	**Subjects age (y) (mean/*SD*)**	**Sex (No. of females/males)**	**No. (%) CPA-positive**	**No. (%) qPCR-positive**	**No. (%) rK39-positive**	**No. (%) DAT-positive**
CVL	14	45 ± 10	5/9	14 (100)	3 (21)	7 (50)	8 (57)
Active VL	12	45 ± 12	5/7	0	12 (100)	8 (67)	10 (83)
AS-Li	57	42 ± 6	15/42	57 (100)	0	0	0
NVL-Li	50	43 ± 12	15/35	0	0	0	0
AS-Ld	12	38 ± 10	5/7	nd	0	11 (91.7)	12 (100)
NVL-Ld	13	33 ± 8	3/10	nd	0	0	0

*VL, visceral leishmaniasis; CVL, cured of visceral leishmaniasis; AS-Li, asymptomatic subject from L. infantum area; AS-Ld, asymptomatic subject from L. donovani area; NVL-Li, negative control from L. infantum area; NVL-Ld, negative control from L. donovani area; CPA, cell proliferation assay; DAT, direct agglutination test; Nd, not determined*.

### Ethics statement

Recruitment and sample collection was performed in accordance with guidelines for good clinical practice. The study was approved by the *Hospital de Fuenlabrada* Ethics and Research Committee, and by the Ethical Review Committee of the International Centre for Diarrhoeal Disease Research, Bangladesh. All study subjects provided their informed written consent to be included.

### rK39 immunochromatographic test

The assay was performed using the dipstick format Kalazar Detect Rapid test (InBIOS International, Seattle, WA). Antibody detection was performed with plasma samples according to the manufacturer's instructions.

### Direct agglutination test

DAT was performed using freeze-dried antigen (ITMA-DAT/VL; Prince Leopold Institute of Tropical Medicine, Antwerp, Belgium) following the manufacturer's recommendations. Serum samples with a titre of 1:3200 were considered positive.

### DNA extraction and real-time PCR

DNA was extracted from 100 μl of peripheral blood by conventional phenol-chloroform extraction and eluted in 100 μl sterile distilled water, as previously described (Cunha et al., 2013). Real-time PCR (qPCR) was then performed to detect leishmanial DNA using 4 μl of the extracted DNA (Cruz et al., 2013) and primers R223 (1,000 nM) and R333 (500 nM; Sigma-Aldrich; both for the small subunit rRNA gene), employing the LightCycler FastStart DNA Master SYBR Green I kit (Roche Applied Science).

### Preparation of soluble *L. infantum* antigen for stimulation of whole blood collected from subjects

*L. infantum* antigen extract was prepared from stationary phase promastigote cultures (JPC strain, MCAN/ES/98/LLM-722) as previously described (Carrillo et al., 2015). Briefly, parasites resuspended in lysis buffer (50 mM Tris/5 mM EDTA/HCl, pH 7) were subjected to three rapid freeze/thaw cycles followed by three 20 s 40 W pulses with a sonicator. Two consecutive 27,000 g centrifugations for 20 min at 4°C were then performed, and the supernatants collected, aliquoted, and stored at −80°C until use. Protein quantification was performed using the Bradford method employing the Bio-Rad Protein Assay kit (Bio-Rad, USA).

### Whole blood stimulation assay

Whole blood samples were stimulated as previously described (Carrillo et al., 2015; Ibarra-Meneses et al., 2016). Briefly, for each sample, an aliquot of blood (500 μL) was placed on its own in a tube (negative control), and another in a tube containing 10 μg/ml SLA, and both were incubated at 37°C for 24 h. After centrifugation at 2,000 g for 10 min, the supernatants were collected and stored at −20°C for cytokine/chemokine analysis.

### Cytometric quantification of cytokines/chemokines

IP-10, MIG, IL-2, and IFN-γ were quantified in 50 μl of plasma from SLA-stimulated whole blood using the BD Cytometric Bead Array Human Flex Set (Becton Dickinson Biosciences, USA) following the manufacturer's instructions. Briefly, 50 μl of the plasma of each subject was incubated for 1 h at room temperature with 50 μl of capture beads. After incubation, 50 μl of the detection antibody was added and mixture placed 2 h at room temperature. Data were acquired using a FACSCalibur flow cytometer and analyzed using the Flow Cytometric Analysis Program Array (Becton Dickinson Biosciences, USA). Results for each chemokine/cytokine were expressed as the difference between the SLA-stimulated and control plasma concentrations in pg/ml.

### Statistical analyses

Cytokine and chemokine concentrations were compared using the Mann–Whitney *U*-test. The cut-off for each analyte was determined by calculating the area under the receiver operating characteristic (ROC) curve (AUC) and the 95% confidence interval (CI). Significance was set at *p* ≤ 0.05. Analyte concentrations before and after treatment were compared using the Wilcoxon paired *t*-test. All calculations were performed using GraphPad Prism 7.0 software (GraphPad Software, USA).

## Results

### IP-10 is a good marker of contact with visceralizing *Leishmania* species, and MIG a good marker of contact with *L. donovani*

**Figure 1:**
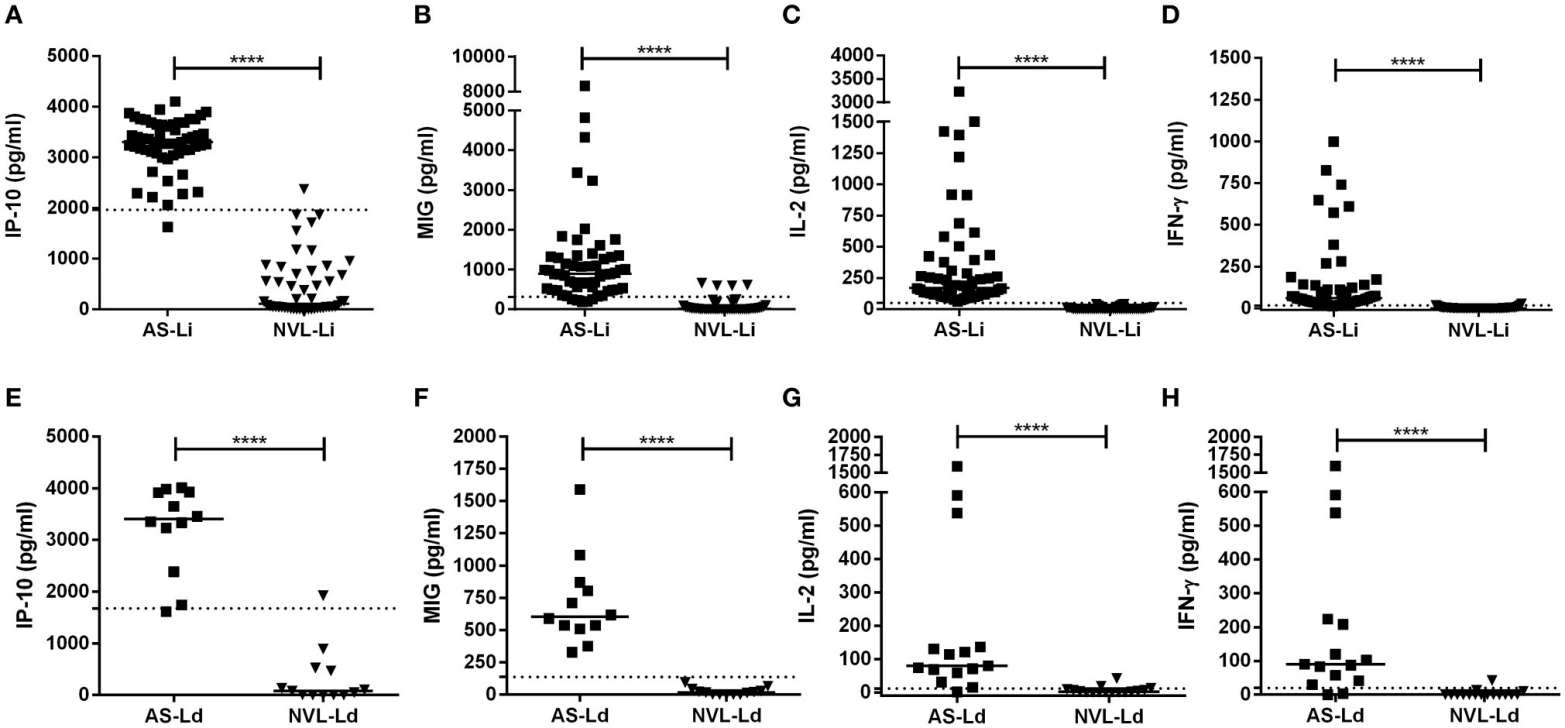
**IP-10, MIG, IL-2, and IFN-γ concentrations in SLA-stimulated whole blood from asymptomatic subjects and negative controls from the *L. infantum* (Fuenlabrada, Spain) (A–D)** and *L. donovani* (Mymensingh, Bangladesh) **(E–H)** areas. Each dot represents one individual; horizontal lines and error bars represent median values. The non-parametric Mann–Whitney *U*-test was used to compare the differences between groups. ^****^p < 0.0001.

IP-10, MIG, IL-2, and IFN-γ were analyzed in blood (SLA-stimulated) taken from subjects exposed to *L. infantum* and *L. donovani* infection (Figure 1). In asymptomatic individuals from the corresponding areas, all three analytes were found in much higher concentrations than in the respective negative controls (Figures 1A–H, respectively). The median IP-10 concentrations of the AS-Li and AS-Ld subjects were similar (3,303 pg/ml compared to 3,406 pg/ml, respectively), and only slight differences were observed in the production of MIG (892 pg/ml in AS-Li subjects and 604 pg/ml in AS-Ld subjects) and IFN-γ (60.07 pg/ml in AS-Li subjects and 89.94 pg/ml in AS-Ld subjects). However, a large difference was detected in the IL-2 concentration of the AS-Li and AS-Ld subjects (172.2 and 79.76 pg/ml, respectively).

The asymptomatic subjects from Bangladesh were seropositive for *L. donovani*, while the asymptomatic subjects from Spain were seronegative for *L. infantum* (Table 1). qPCR returned negative results for all blood samples.

**Figure 2:**
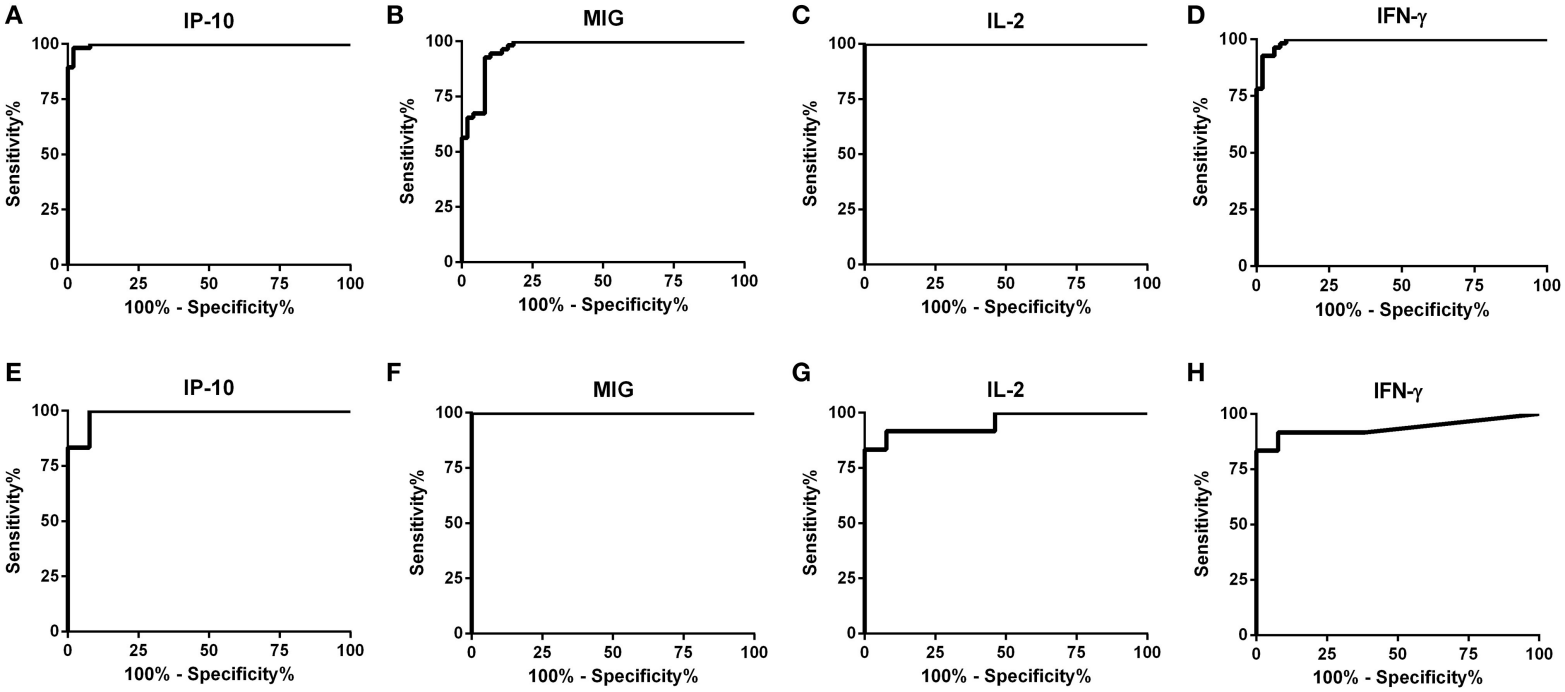
**ROC curve analyses for IP-10, MIG, IL-2, and IFN-γ in SLA-stimulated whole blood from subjects exposed to *L. infantum* (A–D)** and *L. donovani* **(E–H)**.

The detection accuracy for each analyte was determined by ROC analyses (Figure 2). The detection performance of IFN-γ, 52 of 55 subjects were identified as AS-Li while 10 of 12 subjects were found as AS-Ld (Table 2). IL-2 accuracy was 100% (55/55) and 83.33% (10/12) for AS-Li and AS-Ld, respectively. IP-10 resulted to be a good biomarker to detect asymptomatic subjects from both areas of study. The AUC for IP-10 was 0.9939 for the AS-Li subjects and 0.9872 for the AS-Ld subjects. IP-10 was able to identify 54 of 55 and 11 of 12 subjects who were infected with *L. infantum* or *L. donovani*, respectively. In addition, MIG turned out to be the best biomarker to identify asymptomatic subjects from *L. donovani* area. The AUC for MIG was 1.000, and correctly identified 100% (12/12) of asymptomatic subjects. All of the AUC-values were >0.9300 and significantly different (Table 2).

**Table 2:** **Detection accuracy from asymptomatic subjects by using WBA**.

	***L. infantum***					***L. donovani***				
**Analytes**	**AUC (95%CI)**	**Cut-off**	**Se (%)**	**Sp (%)**	**Recognition**	**AUC (95%CI)**	**Cut-off**	**Se (%)**	**Sp (%)**	**Recognition**
IFN-γ	0.9915 (0.980–1.000)	17.23	94.74	98.08	52/55	0.9359 (0.820–1.000)	20.11	83.33	100	10/12
IL-2	1.0000 (1.000–1.000)	50.37	100	100	55/55	0.9551 (0.874–1.000)	16.57	83.33	92.31	10/12
IP-10	0.9939 (0.985–1.000)	1,965	98.25	98.00	54/55	0.9872 (0.954–1.000)	1,678	91.67	92.31	11/12
MIG	0.9659 (0.935–0.997)	312.6	92.73	91.84	51/55	1.0000 (1.000–1.000)	138.1	100	100	12/12

*AUC, Area under the curve; Se, sensitivity; Sp, specificity*.

### IFN-γ, IP-10, and MIG are potential markers for checking the effectiveness of treatment in patients with *L. infantum*-induced VL

**Figure 3:**
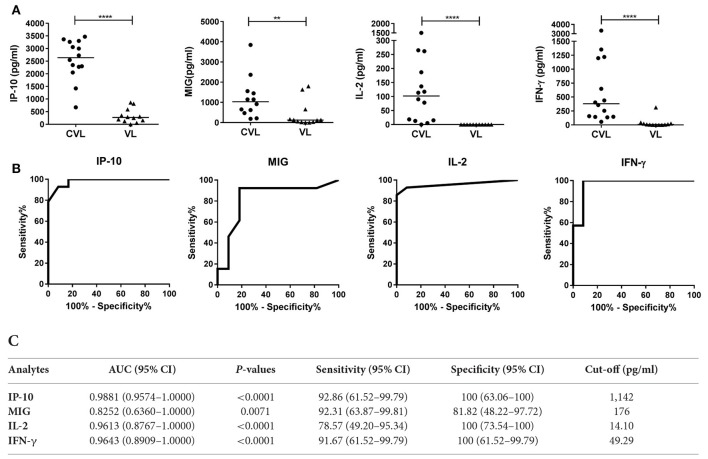
**IP-10, MIG, IL-2, and IFN-γ concentrations in whole blood (after stimulation with SLA) from CVL and active VL patients from the *L. infantum* area**. Whole blood from 14 CVL and 12 active VL subjects was stimulated for 24 h with SLA. Analyte concentrations (pg/ml) were obtained by subtracting the values for the negative control plasma from the values for the SLA-stimulated plasma. **(A)** The horizontal line for the individual biomarkers indicates the median value. **(B)** ROC curve analyses for IP-10, MIG, IL-2, and IFN-γ in blood (SLA-stimulated) from CVL subjects (*n* = 14) at 6 month of follow-up. **(C)** AUC, sensitivity, and specificity values. ^**^P < 0.01, ^****^P < 0.0001.

IP-10, MIG, IL-2, and IFN-γ concentrations were determined in blood (after stimulation with SLA) from 14 CVL and 12 active VL subjects from the *L. infantum* area (Figure 3). The blood of the CVL subjects produced significantly more IP-10, MIG, IL-2, and IFN-γ (median IP-10: 2,638 pg/ml; MIG: 1,033 pg/ml; IL-2: 102 pg/ml; IFN-γ: 380 pg/ml) than did that of the active VL subjects (IP-10: 270.1 pg/ml; MIG: 123.1 pg/ml; IL-2: 0.00 pg/ml; IFN-γ: 7.72 pg/ml). No IL-2 was detected in the SLA-stimulated blood of the active VL patients.

At 6 months of follow-up, the AUCs for IP-10, MIG, IL-2, and IFN-γ were 0.9881 (95%CI: 0.9574–1.000), 0.8252 (95%CI: 0.6360–1.000), 0.9613 (95%CI: 0.8767–1.0000), and 0.9643 (95%CI: 0.8909–1.0000), respectively (Figure 3C).

**Figure 4:**
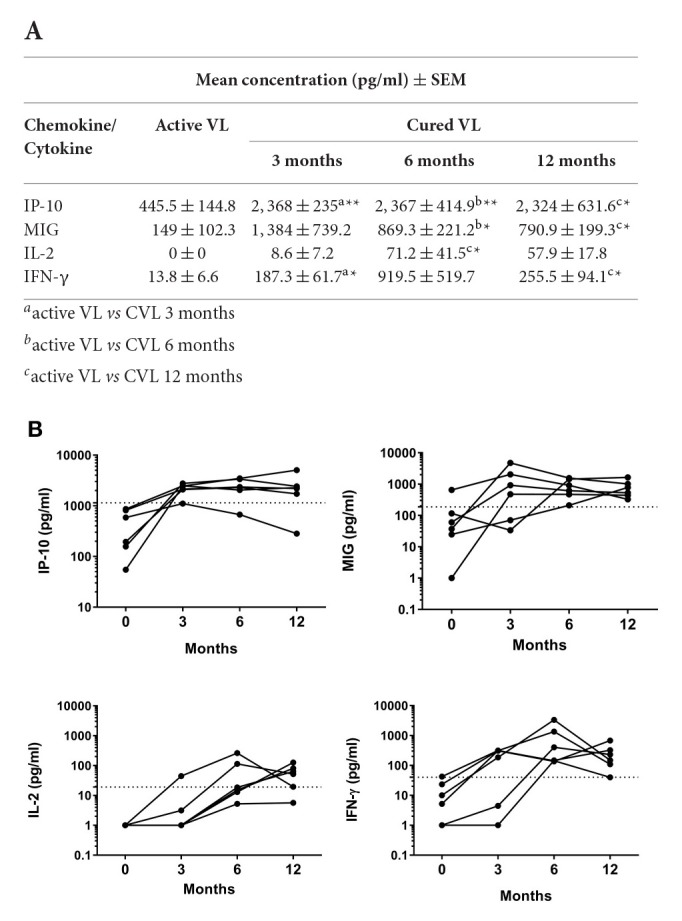
**Changes in IP-10, MIG, IL-2 and IFN-γ in whole blood (SLA-stimulated) following the treatment of patients with active *L. infantum* infection**. **(A)** Mean (SEM) IP-10, MIG, IL-2, and IFN-γ concentrations at 0, 3, 6, and 12 months. **(B)** Cytokine/chemokine concentration before and after VL treatment. The cut-off for cured VL (CVL) is marked with a dashed line. Comparisons of means were made using the Wilcoxon paired *t*-test. ^*^P < 0.05; ^**^P < 0.01.

The chemokines/cytokines in the blood (SLA-stimulated) of the six followed subjects with active VL were also quantified over the latters' treatment period. Significant increases in IP-10, MIG, IL-2, and IFN-γ were seen at month 0 compared to non-infected subjects (Table S1). The concentrations of these chemokines/cytokines rose after treatment, with values remaining higher at 12 months (Figure 4A). The highest IP-10 (maximum mean 2,368 pg/ml; *P* = 0.0026) and MIG (maximum mean 1,384 pg/ml; *p* = 0.0639) concentrations were detected during the first months of follow-up (Figure 4A). Increases in IL-2 and IFN-γ were also seen (maximum mean 71.2 pg/ml, *p* = 0.0108 and 919.5 pg/ml; *p* = 0.0105, respectively), but were slower compared to those seen for the chemokines studied (Figure 4A). The calculated cut-offs (Figure 3C) showed SLA-induced IFN-γ and MIG to distinguish between cured status and active disease status at 6 months in 6/6 patients (100%); IP-10 distinguished in 5/6 patients, and IL-2 did so in 4/6 (Figure 4B).

## Discussion

Parasitological, molecular, and serological diagnostic tools are used to detect asymptomatic subjects in *Leishmania*-endemic areas. However, in the present study, all the asymptomatic subjects were qPCR-negative, and while the rK39/DAT test was able to detect asymptomatic subjects infected with *L. donovani*, it could not detect those infected with *L. infantum*. The combination of these tests with an assay to detect cell-mediated immunity against *Leishmania* might be the best option when trying to determine the real prevalence of asymptomatic status (Gadisa et al., 2012), and in fact the asymptomatic subjects from the *L. infantum* area were all found to be CPA-positive. However, the CPA test is laborious and difficult to run in the field—which is why it was not performed in the *L. donovani* area. The SLA-stimulated whole blood assay, when used in the detection of the new markers identified in the present work, would appear to offer a promising alternative for detecting asymptomatic subjects in both *L. infantum*- and *L. donovani*-affected areas—including in the field.

We previously reported IL-2 to be the most sensitive and specific biomarker for asymptomatic subjects in *L. infantum* areas (Ibarra-Meneses et al., 2016). Although the present results also show IL-2 to be valuable in this respect, it was not as useful for detecting asymptomatic subjects in the present *L. donovani* area; indeed, MIG and IP-10 performed much better. Differences found in the IL-2 levels between AS-Li and AS-Ld might be related with a remote or recent infection, respectively, as described in tuberculosis (Krummel et al., 2010). Longitudinal studies with larger numbers of asymptomatic subjects from different endemic areas and characteristic of the cohorts will be needed for a further exploration and clarification of this or other hypothesis. MIG also showed differences as a marker to identify asymptomatic individuals from both endemic areas, being 100% sensitive and specific for those infected with *L. donovani*. The high sensitivity and specificity of IP-10 show it can be used as a biomarker for identifying asymptomatic individuals living in both *L. donovani* and *L. infantum* areas. IP-10 and MIG have both been described as potential biomarkers for assessing latent tuberculosis (Lighter et al., 2009; Rubbo et al., 2012), but this is the first study of these chemokines as biomarkers for use with SLA-stimulated blood.

IFN-γ in SLA-stimulated whole blood has previously been suggested as a biomarker for identifying patients cured of leishmaniasis. Adem et al. (2016) reported antigen-specific IFN-γ to be gradually but significantly increased at 3 and 6 months after the start of successful VL treatment in Ethiopia (Adem et al., 2016). In a previous study on patients with *L. infantum*-induced VL in Spain, we described results comparable to those recorded by Adem et al. for IFN-γ and IL-2 at 3 months after the start of treatment (Ibarra-Meneses et al., 2016). However, Singh et al. (2012) reported that IFN-γ levels in SLA-stimulated blood were similar in Indian patients with active VL and in cured patients at least 6 months after successful treatment (Singh et al., 2012). We hypothesize that one of the factors that could be influencing these results is the time when whole blood assay was performed after cure. Present results and others (Adem et al., 2016; Ibarra-Meneses et al., 2016) found that IFN-γ increased at 3 and 6 months after start of the treatment, but we described here that mean levels of IFN-γ at 12 months experimented a three-fold decrease regarding to 6 months. In the report from India where IFN-γ levels were similar in active and cured subjects, whole blood assay was performed at least 6 months after successful treatment but the time is not further specified (Singh et al., 2012). Due to small size of our cohort, further investigation into the influence of the time after cure to perform whole blood assay for the monitoring of immunity is needed. However, other epidemiological factors of the endemic area and nutritional condition of the subjects together with the treatment received may be influencing these results.

The present work is the first to use a whole blood assay to monitor the same individuals from the time of active disease through to their cure, and to study the immune response over a period of 1 year. The results show IFN-γ, but not IL-2, to be very useful for monitoring the effectiveness of treatment for VL with liposomal amphotericin B. The results also reveal IP-10 and MIG to be excellent markers of cure in patients with *L. infantum* infection. A single previous report records MIG and IP-10 as biomarkers of cure in *non*-SLA stimulated plasma; IFN-γ, IL-12p40, IL-18, IL-15, IP-10, and MIG concentrations were all markedly elevated in patients with active VL compared to healthy controls (Hailu et al., 2004). IP-10 and MIG are also reported to be markers of recovery from tuberculosis (Azzurri et al., 2005; de Steenwinkel et al., 2012). The present tests should be repeated in immunocompetent individuals from other endemic areas before the accuracy of these chemokines as a marker of “cured” status can be fully established. In addition, studies including subjects treated with other drugs than amphotericin B are needed before concluding the global use of these markers to identify patient cure. Assessments should also be made in the pediatric population. Cell-mediated immunity tests have recently been proposed for the follow-up of patients co-infected with HIV/*Leishmania* (Castro et al., 2016). Since IP-10 identifies HIV/tuberculosis-infected patients, despite their low CD4 counts (Azzurri et al., 2005; Kassa et al., 2016), it might be used to monitor for relapses of leishmaniasis in HIV/*Leishmania*-infected patients.

In conclusion, the present work shows that IP-10 and MIG concentrations can be used to identify asymptomatic subjects infected with *L. infantum* or *L. donovani*. Further, IFN-γ, MIG and IP-10 can be used as markers of cure in immunocompetent patients treated for *L. infantum*-induced VL. The higher concentrations of IP-10 and MIG than IFN-γ and IL-2 might be easy to detect with simple-to-use platforms (such as ELISA- and immunochromatographic-based assays) that could be employed in the field. This minimally invasive, non-sensitizing, simple assay could be of great value in epidemiological studies performed in the field. The better identification of asymptomatic and cured subjects would help in the control of VL. Our group is currently validating the use of IFN-γ, IL-2, and IP-10 as biomarkers for quantifying the prevalence of asymptomatic status in a cross-sectional study in Madrid (Spain). A study to assess the potential of IFN-γ, IP-10, and MIG as biomarkers in the follow-up of treated patients is underway in India.

## Summary

This paper reports IP-10 and MIG in SLA-stimulated whole blood to be potential markers of subjects asymptomatic for *L. infantum* and *L. donovani* infection. It also establishes proof of concept that plasma IP-10 may be used as a marker of treatment response and cure in *L. infantum*-induced visceral leishmaniasis. The very large area under the receiver operating characteristic curves for IP-10 and MIG provides good evidence of the latters' ability to identify asymptomatic subjects and to act as markers of the cellular immune response after treatment. These chemokines appear in much greater concentration than IFN-γ, and are therefore more robust markers—as described for them regarding the diagnosis of tuberculosis. Testing for multiple biomarkers might allow the results provided by the main marker to be confirmed.

## Author contributions

AI, JA, JM, and EC designed the study; AI, PG, FH, and RC performed experiments; AI analyzed data; AI and EC wrote the manuscript; AI, DM, JA, JM, and EC interpreted the data.

### Conflict of interest statement

The authors declare that the research was conducted in the absence of any commercial or financial relationships that could be construed as a potential conflict of interest.
