# Supplementary material for: IFN-γ, IL-2, IP-10, and MIG as Biomarkers of Exposure to Leishmania spp., and of Cure in Human Visceral Leishmaniasis
Source: Front Cell Infect Microbiol. 2017 May 31;7:200. doi: 10.3389/fcimb.2017.00200 (PMC5449718; doi:10.3389/fcimb.2017.00200)
Supplement: Supplementary file 1 [file Table1.DOCX]

**Supplementary table**

**S1.** Area under the curve, sensitivity and specificity values from CVL/NVL-Li

| **Analytes** | **AUC (95% CI)** | **P value** | **Sensitivity (95% CI)** | **Specificity (95% CI)** | **Cut-off (pg/ml)** |
| --- | --- | --- | --- | --- | --- |
|  |  |  |  |  |  |
| **IP-10** | 0.9701 (0.9225-1.0000) | <0.0001 | 93.48 (82.10-98.63) | 87.5 (47.35-99.68) | 1320 |
| **MIG** | 0.8485 (0.6478-1.0000) | 0.0025 | 81.82 (64.54-93.02) | 87.5 (47.35-99.68) | 202.6 |
| **IL-2** | 0.7948 (0.7981-1.0000) | 0.0082 | 80.43 (72.07-94.70) | 75 (34.91-96.81) | 12.82 |
| **IFN-γ** | 1.0000 (1.0000-1.0000) | <0.0001 | 100 (92.29-100) | 100 (63.06-100) | 41.28 |
